# Introducing an interesting and novel strategy based on exploiting first-order advantage from spectrofluorimetric data for monitoring three toxic metals in living cells

**DOI:** 10.1016/j.toxrep.2022.03.049

**Published:** 2022-04-01

**Authors:** Vali Akbari, Elaheh Jamasbi, Shahla Korani, Hamid-Reza Mohammadi-Motlagh, Ghobad Mohammadi, Ali R. Jalalvand

**Affiliations:** a Research Center of Oils and Fats, Research Institute for Health Technology, Kermanshah University of Medical Sciences, Kermanshah, Iran; b Medical Biology Research Center, Health Technology Institute, Kermanshah University of Medical Sciences, Kermanshah, Iran

**Keywords:** Lead, Zinc, Cadmium, Determination, Chemometrics, HeLa Cells

## Abstract

In this work, we did our best to develop a novel and interesting analytical method based on coupling of spectrofluorimetry with first-order multivariate calibration techniques for simultaneous determination of lead (Pd), zinc (Zn) and cadmium (Cd) in HeLa cells. To achieve this goal, quenching of the emission of graphene (GR) was individually investigated in the presence of Pb, Zn and Cd and then, according to the linear ranges obtained from individual calibration graphs, a multivariate calibration model was developed based on modeling of the quenching of the emission of GR in the presence of the mixtures of Pb, Zn and Cd. First-order multivariate calibration models were constructed by partial least squares (PLS), principal component regression (PCR), orthogonal signal correction-PLS (OSC-PLS), continuum power regression (CPR), robust continuum regression (RCR) and partial robust M-regression (PRM) and their performances were evaluated and statistically compared. Finally, the OSC-PLS was chosen as the best model with the best practical performance for analytical purposes.

## Highlights

• The GR was uptaken by the HeLa cells and then, the cells uptook Pb, Cd and Zn.
• Individual and multivariate calibration models were developed by the use of several first-order algorithms.
• Comparing the performance of the models was performed and the OSC-PLS showed the best performance.
• Performance of the OSC-PLS was compared with a reference method.
• Our records showed that the OSC-PLS had a comparable performance with the reference method.

## Graphical Abstract

**Figure:**
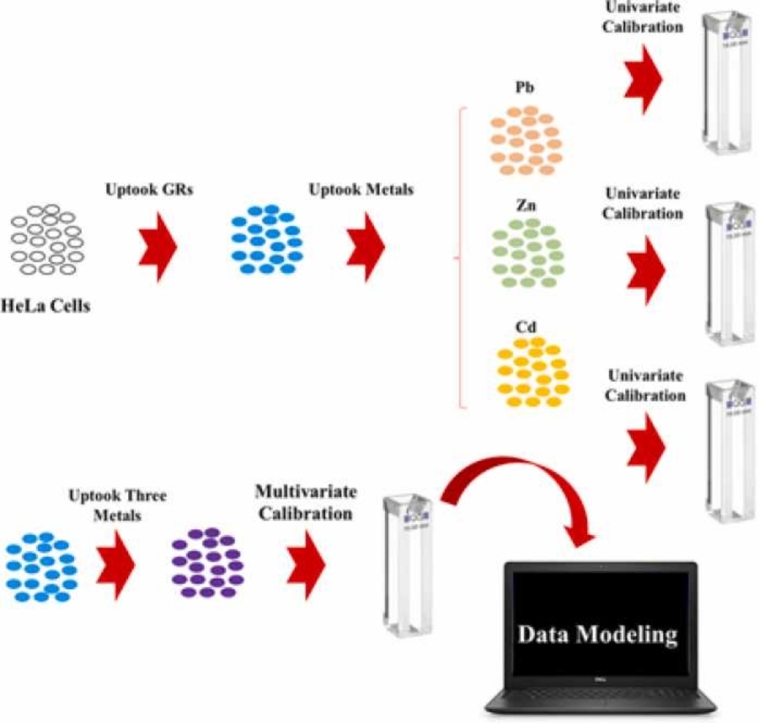

## 1 Introduction

Nanomaterials have strange and valuable properties compared with bulk materials and because of that are widely used for different purposes especially for sensing purposes [1], [2], [3], [4], [5], [6], [7], [8], [9], [10]. Graphene is a two-dimensional carbon nanomaterial which is not only a flexible structure but also is a robust structure which make it to be very useful for different applications [1]. The graphene can be existed in different structures such as graphene oxide, graphene quantum dots and graphene nanoplatelets [2], [3]. The graphene because of having good electrical, thermal and optical properties, has a great potential for application to developing transistors [2], [4], chemical and electrochemical sensors [5] and biological sensors [6]. The graphene has some extra applications in surface coatings for inhibiting corrosions [7], [8] and to reduce wear and friction on sliding metal surfaces [9], [10]. The graphene sheets with lateral dimensions less than one hundred nanometers are called graphene quantum dots (GR) which have new chemical and physical properties such as high stability, good solubility, low toxicity, photoluminescence and excellent biocompatibility.

Heavy metals are existed in the earth's crust but their geochemical cycles and biochemical balance have been significantly affected by human activities. Sometimes, the heavy metals are considered as contaminants which can be hazardous for human health therefore, monitoring of them is important. Lead (Pb) and cadmium (Cd) are heavy metals which are widely and naturally distributed toxic metals. There are some reports on determination of these metals with zinc (Zn) [11]. The Zn is one of the most abundant metals in the human body which is a vital element for growth. There are more than 300 enzymes in human body whose active sites contain the zinc ions and Zn has an important role in synthesis of DNA and RRNA and protein and in cell division as well. Therefore, determination of these three metal ions is interesting and so important. Determination of heavy metals is usually performed by atomic absorption spectroscopy (AAS), inductively coupled plasma, atomic emission spectroscopy, X-ray fluorescence spectroscopy and mass spectroscopy which need expensive instruments which can’t be accessible in most of all of laboratories therefore, developing new analytical methods which are fast, low-cost and accessible is sensible.

HeLa is an immortal cell line which is the most commonly used human cell line in scientific research. The HeLa cell line is durable and prolific which make it to be extremely suitable for scientific research. Therefore in this study, we have used the HeLa cells as a very interesting case for developing a novel analytical method for simultaneous determination of the Pb, Cd and Zn.

**Scheme 1:**
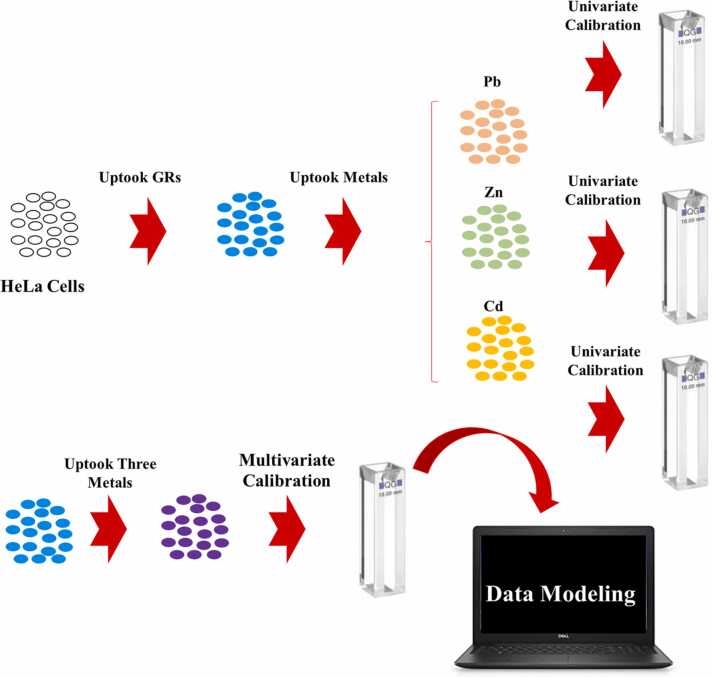
Graphical representation of the steps of project described in this article.

Chemometrics combines chemical data with mathematical and statistical methods to extract useful information which can help the chemists to better justify their observations. Chemometricians have performed different projects by the use of instrumental data [12], [13], [14], [15], [16], [17], [18], [19], [20], [21], [22], [23]. In this project, we are going to couple first-order chemometric multivariate calibration techniques with spectrofluorimetric data to develop a novel analytical method for simultaneous determination of the Pb, Cd and Zn in HeLa cells. To achieve this goal, the GRs were uptaken by HeLa cells and then, Pb, Cd and Zn were individually uptaken and fluorescence quenching of the GRs was recorded in the presence of the metals to obtain individual calibration graphs. Then, a mixture design was used to multivariate calibration of the quenching of the GRs in the presence of Pb, Cd and Zn simultaneously. The spectrofluorimetric responses of the mixtures were modeled by partial least squares (PLS), principal component regression (PCR), orthogonal signal correction-PLS (OSC-PLS), continuum power regression (CPR), robust continuum regression (RCR) and partial robust M-regression (PRM) to build multivariate calibration models and finally, their performance were compared and the best multivariate calibration model was chosen for practical purposes. Schematic representation of the steps described above are shown in Scheme 1.

## 2 Experimental

### 2.1 Chemicals

Trypsin-EDTA, Dulbecco’s modified Eagle’s medium (DMEM/F-12 (1:1)), fetal bovine serum (FBS, 10%), penicillin-streptomycin (PEN-STREP), zinc nitrate hexahydrate, cadmium nitrate tetrahydrate and Pb(NO_3_)_2_ were purchased from Sigma. Commercial Pb, Cd and Zn standards (1 g l^−1^) were prepared from Merck. Graphene quantum dots (blue luminescent) were purchased from Sigma-Aldrich. The other chemicals which were needed for doing this project were available in archive of our laboratory which had been purchased from Sigma or Merck. Doubly distilled water was used wherever water was needed. A phosphate buffer solution (PBS, 0.01 M) was prepared from Na_2_HPO_4_ and its pH was adjusted at 7.4 by the use of H_3_PO_4_ and NaOH.

### 2.2 Instruments and software

Spectrofluorimetric data were recorded by a Cary Varian spectrofluorimeter equipped with a quartz cell (1 cm length path). First-order multivariate calibration algorithms including PLS, PCR, OSC-PLS, CPR, RCR, PRM, smoothing of the data and elliptical joint confidence region (EJCR) were run in MATLAB (Version 7.5) by the use of a series of m-files. The first-order multivariate calibration algorithms have been run in MATLAB with the help of PLS-toolbox or TOMCAT. The HeLa cells were prepared from the cell bonk of Kermanshah University of Medical Sciences. Then, the flask was transferred into a culture room where a deep-freezer (−80 °C), a memmert incubator, a JTLV CZS hood and a Motic microscope were existed for cell culturing. pH adjustments were performed by a Jenway pH meter 3510. Performance of the developed methodology was compared with the results of an Agilent atomic absorption spectrometer as reference method (AAS). Operating conditions for the AAS were: PMT voltage (450 V), slit width (0.40 nm), lamp current (9.0 mA), sample volume (20 µl), purging gas (argon), sample injection replicates (2) and measurement (peak height). All the calculations which were needed for data processing were performed on a Dell XPS laptop.

### 2.3 Procedure

Dispersion of the HeLa cells were performed in DMEM + FBS (10%) + PEN-STREP (1%) and seeded on five confocal dishes and then, they were incubated at an humidified atmosphere (5% CO_2_ +95% air) at 37 °C during a day (24 h). For uptaking the GR, 100 ng mL^−1^ GR was added to different culture dishes and incubated at different times and then, the cells were washed with PBS (0.01 M, pH 7.4) and left to be in the PBS.

For simultaneous determination of Pb, Cd and Zn in HeLa cells, the seeded cells were allowed to grow during a day (24 h) and 1 mL DMEM having 1300 ng mL^−1^ GR was used to replacing the culture medium of each dish and for uptaking the GR, the procedure was continued by incubating the dishes in an incubator for 2 h. Afterwards, the extra amounts of GR were removed by washing the dishes with the PBS for three times. Then, 1 mL DMEM having different concentrations of Pb, Cd and Zn (for all the three metals: 700–1600 ng mL^−1^, with an interval of 100 ng mL^−1^) were added to the dishes. The cells were further incubated for 2 h and washed with the PBS for three times and kept in the PBS. Spectrofluorimetric monitoring of the Pb, Cd and Zn was performed by excitation at 405 nm. For performing background correction on the data, the control cells which had not been incubated with GR (didn’t have any GR) was prepared. The procedures described above were continued by digestion of the treated and control cells with trypsin and then, the cells were kept in the PBS. Afterwards, the cells were counted, broken by ultrasonic and centrifuged. Finally, the supernatant of cells were measured spectrofluorimetrically.

### 2.4 Theoretical details in brief

In this work, we are going to develop a novel spectrofluorimetric method assisted by chemometric methods which will enable us to simultaneous determine Pb, Cd and Zn in living cells. Data treatment and development of multivariate calibration models must be very carefully performed to achieve the final goal. Prior to data modeling, all the spectrofluorimetric data were treated according to the following equation [24]:

(1) $$F_{\mathrm{Cor}}=F_{\mathrm{Obs}}\exp\left[\frac{A_{\mathrm{ex}}+A_{\mathrm{em}}}{2}\right]$$

All the data used in this work after passing this correction step was used for the next steps. Emission of the control cells was subtracted from the emission of the all of the cells and the corrected emissions were used for developing multivariate calibration models. Background correction was performed on the whole of data by subtracting emission of the control cells from emission of the whole of sets. Performance of the calibration models will be compared by the use of the following equations (*RMSEP*: root mean square error of prediction and *REP*: relative error of prediction):

(2) $$\mathrm{RMSEP}=\sqrt{\frac{\sum_{1}^{n}{\left(y_{\mathrm{pred}}-y_{\mathrm{act}}\right)}^{2}}{n}}$$

(3) $$\mathrm{REP}\left(\%\right)=\frac{100}{y_{\mathrm{mean}}}\sqrt{\frac{1}{n}\sum_{i=1}^{n}{\left(y_{\mathrm{pred}}-y_{\mathrm{act}}\right)}^{2}}$$

where *y*_*act*_ and *y*_pred_ are nominal and predicted concentrations, respectively, and *y*_mean_ is the mean of the nominal concentrations. *n* are the number of samples in the validation set. Precision and accuracy of the developed calibration models will be compared according to the ellipses of the EJCR as well. Univariate calibrations and multivariate calibration and validation sets were performed in internal medium of the cells and by digestion of the cells with trypsin, the medium was extracted. This is a very important advantage which causes having a same medium for calibration and validation of the method which can help us for exploiting first-order advantage.

## 3 Results and discussion

### 3.1 Individual calibration graphs

Generally, developing a novel analytical method needs a calibration step by which an instrumental signal is connected with concentration of the analyte of the interest. Therefore, in this project, at the first step, we must calibrate the spectrofluorimetric response of the GR with concentration of the Pb, Cd and Zn. This goal can be achieved by recording spectrofluorimetric responses of the GR in the presence of Pb, Cd and Zn individually. Building the individual calibration curves needed some complicated steps which will be expanded in this section.

**Fig. 1:**
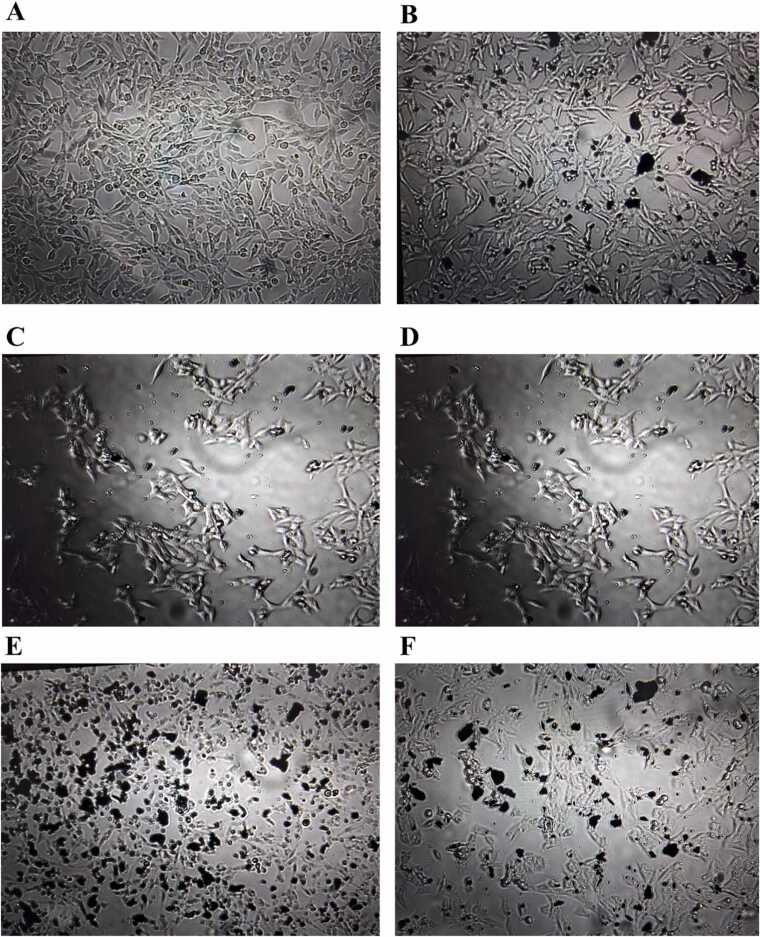
The images related to: (A) the control cells, (B) the cells which uptook 1300 ng mL^−1^ GRs, (C) the cells which uptook 1300 ng mL^−1^ GRs and 500 ng mL^−1^ Pb, (D) the cells which uptook 1300 ng mL^−1^ GRs and 500 ng mL^−1^ Zn, (E) the cells which uptook 1300 ng mL^−1^ GRs and 500 ng mL^−1^ Cd, and (F) the cells which uptook 1300 ng mL^−1^ GRs, 500 ng mL^−1^ Pb, 500 ng mL^−1^ Zn and 500 ng mL^−1^ Cd.

The HeLa cells which had uptaken 1300 ng mL^−1^ GR, were used to uptaking different concentrations of Pb, Cd and Zn from 700 to 1600 ng mL^−1^. The images related to the control cells, the cells which uptook GRs, the cells which uptook GRs and Pb, the cells which uptook GRs and Zn, the cells which uptook GRs and Cd and the cells having all of the three metals are shown in Fig. 1A-F, respectively. As can be seen, obvious variations were observed among the images which confirmed successful uptaking GR and metals in HeLa cells.

**Fig. 2:**
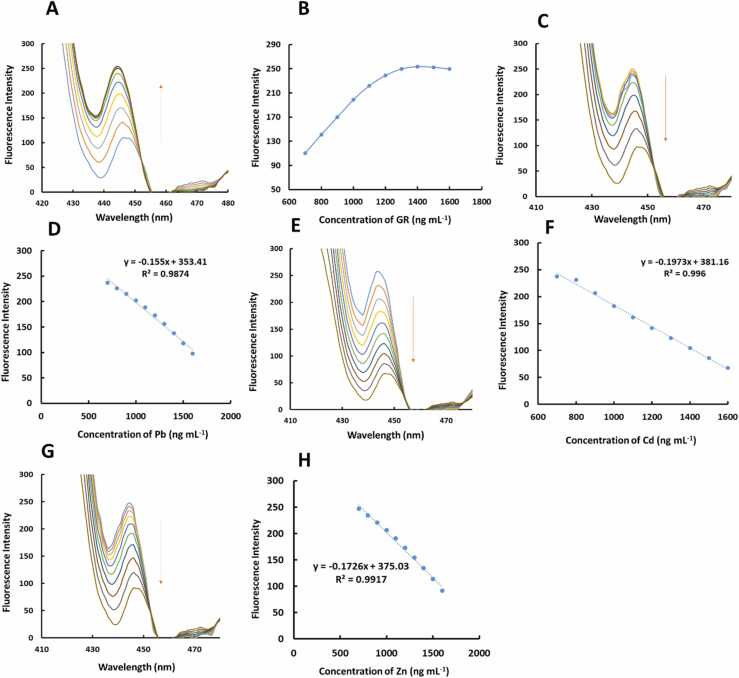
(A) Emission spectra obtained from recording spectrofluorimetric responses of the broken cells having different concentrations of the GR and (B) variation of the maximum of the spectrofluorimetric responses of the broken cells having different concentrations of the GR versus concentration of the GR. (C) Spectrofluorimetric responses of the broken cells having 1300 ng mL^−1^ GR and increasing concentration of the Pb and (D) the calibration graph obtained by the regression of the currents of (C) on concentration of the Pb from 700 to 1600 ng mL^−1^. (E) Spectrofluorimetric responses of the broken cells having 1300 ng mL^−1^ GR and increasing concentration of the Cd and (F) the calibration graph obtained by the regression of the currents of (E) on concentration of the Cd from 700 to 1600 ng mL^−1^. (G) Spectrofluorimetric responses of the broken cells having 1300 ng mL^−1^ GR and increasing concentration of the Zn and (H) the calibration graph obtained by the regression of the currents of (G) on concentration of the Zn from 700 to 1600 ng mL^−1^.

After observation of the appearance of the cells microscopically, the broken cells were monitored spectrofluorimetrically. It should be noted that prior to selection of the optimum concentration of the GR for having the best emission, its concentration was varied and its emission was recorded as the data shown by Fig. 2A. Variation of the emission of the GR versus concentration of the GR is shown by Fig. 2B, and as can be seen, the graph is increased and leveling off which helped us to choose 1300 ng mL^−1^ as the optimum concentration of the GR. Afterwards, the broken cells having GR and Pb, Cd and Zn were monitored individually to build the individual calibration graphs which are shown in Fig. 2C-H. The calibration graphs gave us the linear ranges where emission of the GR was linearly correlated with concentration of the Pb, Cd and Zn which will be used for developing multivariate calibration models.

### 3.2 Multivariate calibrations

**Fig. 3:**
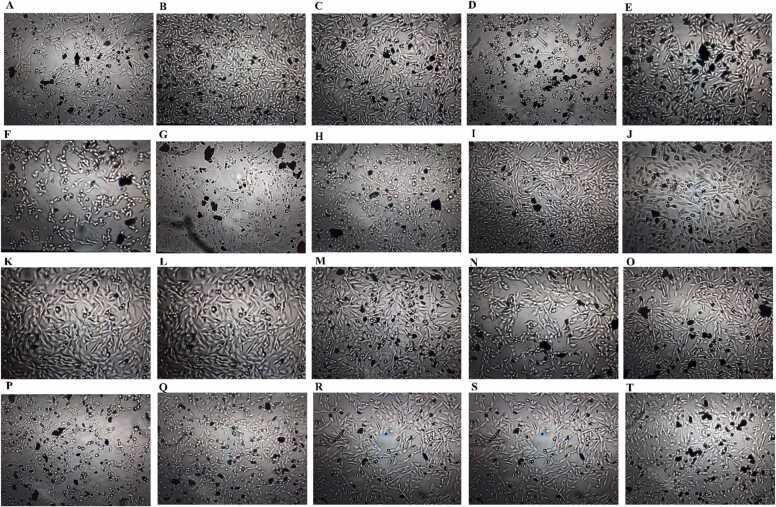
(A)-(J) The images related to the runs (C_1_-C_10_) of the calibration set and (K)-(T) images related to the runs (V_1_-V_10_) of the validation set.

**Fig. 4:**
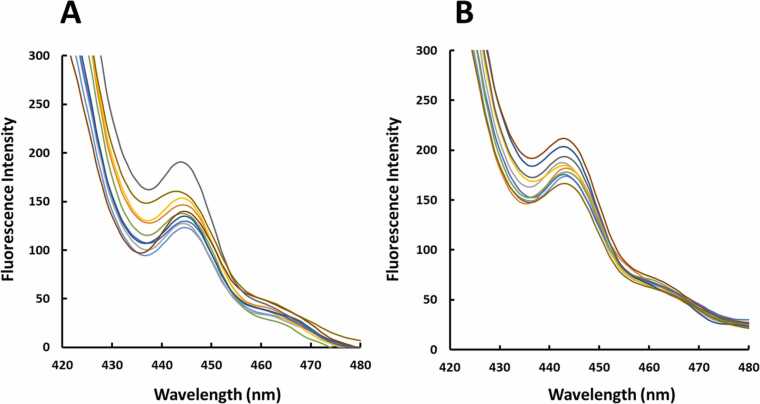
(A) and (B) Spectrofluorimetric responses of the cells related to the calibration and validation set, respectively.

**Fig. 5:**
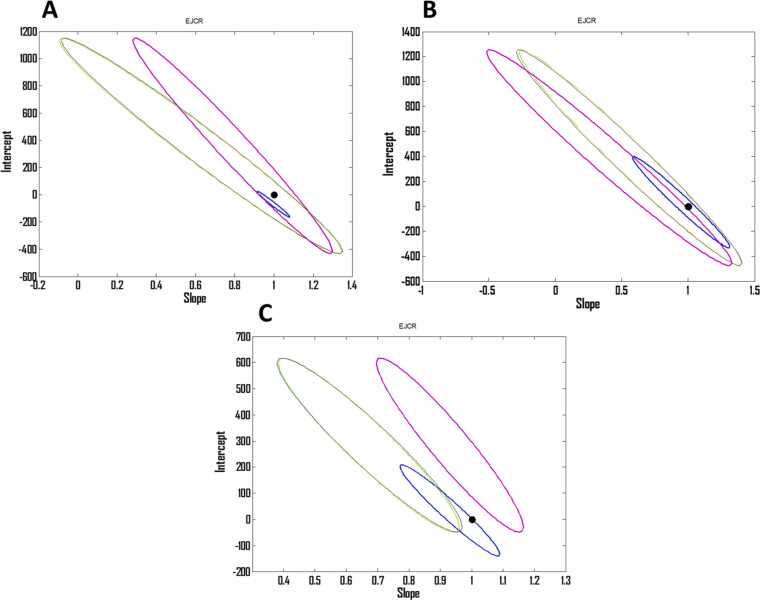
(A), (B) and (C) Ellipses obtained by EJCR related to the prediction of the concentration of Pb, Zn and Cd, respectively. Blue ellipse, pink ellipse, green ellipse, yellow ellipse, black ellipse and red ellipse are related to OSC-PLS, PLS, PRM, CPR, RCR and RCR, respectively. The black point shows the ideal point.

In order to multivariate calibrate the emission of the GR with concentration of Pb, Cd and Zn, a central composite design was developed based on linear ranges obtained from individual calibration graphs. Composition of the calibration set is shown in Table 1. All the cells related to the calibration set had 1300 ng mL^−1^ GR as its optimum concentration where each run had different concentrations of the Pb, Cd and Zn chosen according to the linear ranges obtained from individual calibration graphs. The images taken from the cells related to the calibration set are shown by Fig. 3A-J. The work was continued by the application of PLS, PCR, OSC-PLS, CPR, RCR and PRM to the spectrofluorimetric data recorded for the calibration set which are shown by Fig. 4A. Wherever number of latent variables (LVs) was required, it was determined by leave one our cross validation (LOOCV). Different algorithms used in this study needed some parameters which were optimized as follows: PLS: number of LVs = 3, OSC-PLS: number of LVs = 3, CPR: number of LVs = 3 and power = 1, RCR: number of LVs = 3, percentage of data contamination = 0.1 (PDC) and delta parameter= 0.05 (*δ*) and PRM: number of LVs = 3 and PDC = 0.12. After application of the algorithms and optimization of their parameters and constructing multivariate calibration models, their performance was verified by their application to a validation set having cells with different concentrations of Pb, Cd and Zn whose composition is shown by Table 2. The images taken from the cells related to the validation set are shown by Fig. 3K-T and their spectrofluorimetric responses are shown by Fig. 4A. Application of the constructed multivariate calibration models to the validation set for examination of their performance was performed and the predicted concentrations by different algorithms have been collected in Table 3. By calculating REPs and RMSEPs which are collected in Table 4, it can be clearly seen that OSC-PLS had the best performance among the tested algorithms and their performance obeys from the following order: OSC-PLS>PLS>PRM>RCR~PCR~CPR. For further comparison of different algorithms, their accuracy and precision were compared by the use of EJCR and the results are shown in Fig. 5. The outputs of the EJCR are ellipses whose size is proportional to the precision of the method and falling the ideal point within the ellipse confirms the accuracy of the method. The ellipses related to the application of different algorithms for prediction of Pb, Zn and Cd in the validation set are shown in Fig. 5A, B and C, respectively. Blue ellipse, pink ellipse, green ellipse, yellow ellipse, black ellipse and red ellipse are related to OSC-PLS, PLS, PRM, CPR, RCR and RCR, respectively, and the black point shows the ideal point. Yellow, green, black and red ellipses were fallen to each other and only blue and pink ellipses were apparently different from the other ellipses. According to the results of EJCR, the blue ellipse which was related to OSC-PLS confirmed the best performance which motivated us to select it as the best model for simultaneous determination of the Pb, Zn and Cd.

**Table 1:** Concentrations (ng/mL) of the metals in the calibration set.

Run	Cd	Pb	Zn
C_1_	700	700	700
C_2_	700	1100	1500
C_3_	700	1500	700
C_4_	700	1500	1500
C_5_	1500	700	1100
C_6_	1500	700	1500
C_7_	1500	1500	700
C_8_	1500	1500	1500
C_9_	1100	1100	1100
C_10_	1100	1100	1100

**Table 2:** Concentrations (ng/mL) of the metals in the validation set.

Run	Cd	Pb	Zn
1	800	1000	700
2	1000	1200	1000
3	900	900	1200
4	1200	800	800
5	1600	700	850
6	750	1400	1200
7	800	1300	1300
8	900	950	700
9	1400	1000	950
10	800	1050	900

**Table 3:** Predicted concentrations of the validation set by different algorithms.

Algorithm	Cd	Pb	Zn	Algorithm	Cd	Pb	Zn
PLS	781	1091	781	PCR	647	850	871
	920	1267	930		1192	1321	837
	987	957	1280		1059	1094	1023
	1269	891	856		1376	995	1014
	1659	780	750		1781	894	996
	780	1486	1110		895	1211	1022
	856	1370	1347		621	1500	1508
	960	1001	758		709	750	931
	1480	1090	988		1604	1183	1190
	850	1095	983		991	912	706
Algorithm	Cd	Pb	Zn	Algorithm	Cd	Pb	Zn
OSC-PLS	800	1001	701	CPR	648	851	870
	1001	1200	1001		1190	1320	838
	901	900	1200		1061	1091	1021
	1200	800	800		1375	996	1012
	1601	700	851		1780	895	998
	750	1400	1200		896	1216	1020
	800	1300	1300		621	1505	1506
	900	951	699		708	752	931
	1400	1000	951		1605	1188	1188
	801	1050	902		998	915	704
Algorithm	Cd	Pb	Zn	Algorithm	Cd	Pb	Zn
RCR	651	847	866	PRM	910	890	601
	1180	1311	831		1110	1298	903
	1050	1080	1001		800	1010	1310
	1367	990	1011		1305	698	702
	1760	890	991		1702	800	961
	890	1210	1020		851	1506	1098
	601	1500	1505		975	1081	600
	704	748	931		998	1061	805
	1601	1180	1191		1511	1108	851
	991	910	711		891	1150	802

**Table 4:** The REP and RMSEP values related to the prediction of the validation set by different algorithms.

	PLS	OSC-PLS	PCR	RCR	CPR	PRM
REP(%, Cd)	6.1612	0.0623	17.5396	17.2087	17.6132	11.0881
RMSEP (Cd)	62.5364	0.6325	178.0267	174.6680	178.7736	112.5438
REP(%, Pb)	7.2473	0.0434	17.3320	17.0729	17.3180	11.7868
RMSEP (Pb)	74.6472	0.4472	178.5195	175.8505	178.3752	121.4043
REP(%, Zn)	7.5837	0.0988	20.2468	20.3514	20.2333	25.1745
RMSEP (Zn)	72.8032	0.9487	194.3698	195.3735	194.2395	241.6752

**Fig. 6:**
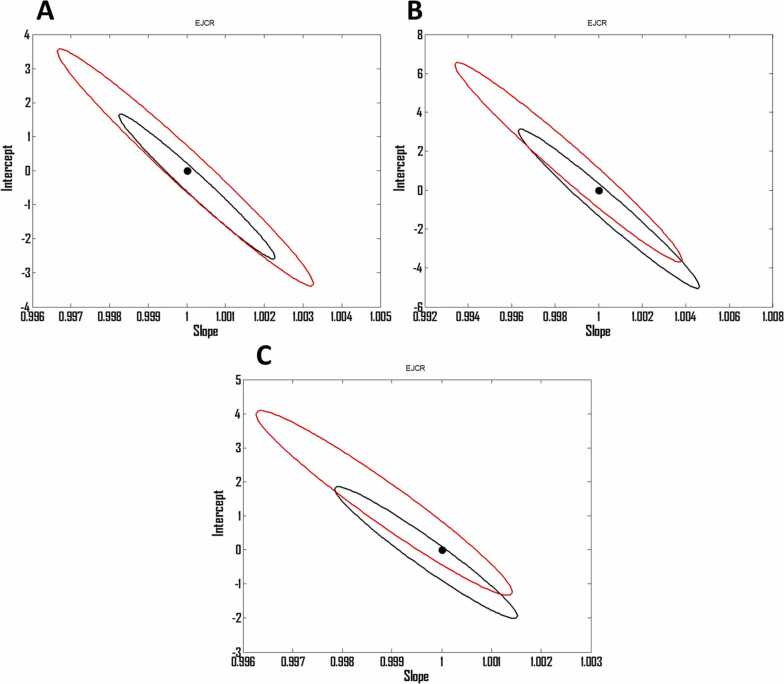
(A), (B) and (C) Ellipses obtained by EJCR related to the prediction of the concentration of Pb, Zn and Cd in validation set, respectively. Black ellipse and red ellipse are related to AAS and OSC-PLS, respectively. The black point shows the ideal point.

In order to further verification of the performance of the spectrofluorimetric method assisted by OSC-PLS, the AAS was applied to the prediction of the concentrations of the validation set as reference method and the results are shown in Table 5. The REPs and RMSEPs are presented in Table 5 as well, and as can be seen, the method showed a good performance. For graphical comparison of the AAS and OSC-PLS by the use of EJCR, their results were fed to MATLAB and the EJCR was run on them and the results are shown in Fig. 6. As can be seen, the AAS (black ellipse) showed better accuracy and precision than OSC-PLS (red ellipse) but, by tacking to account that the OSC-PLS is low-cost, simple and fast method in comparison with the AAS which motivated us to suggest it for practical applications.

**Table 5:** Predicted concentrations of the validation set by the reference method.

	Cd	Pb	Zn
1	800	999.5	699
2	999	1199	1000
3	900	900	1199
4	1201	799	801
5	1601	700	851
6	749	1400	1202
7	799	1300	1300
8	900	951	699
9	1399	1000	949
10	800	1051	899
REP (%, Pb)	0.0633		
RMSEP (Pb)	0.6519		
REP (%, Cd)	0.0763		
RMSEP (Cd)	0.7746		
REP (%, Zn)	0.1093		
RMSEP (Zn)	1.0488

The intra-day precision of the assay was estimated by calculating the relative standard deviation (*RSD*) for the analysis of 800 ng mL^−1^ Pb, Zn and Cd in six replicates which gave us RSDs of 2.08%, 2.15% and 2.11% for Pb, Zn and Cd, respectively. Inter-day precision was determined by the analysis of six replicates 800 ng mL^−1^ Pb, Zn and Cd on three consecutive days which gave us RSDs of 2.34%, 2.28% and 2.21% for Pb, Zn and Cd, respectively. The results obtained for examination of intra-day and inter-day precision confirmed acceptable precisions for the developed methodology.

## 4 Conclusion

In this work, a novel and interesting analytical methodology based on coupling of spectrofluorimetry and chemometrics was developed for simultaneous determination of Pb, Cd and Zn in Hela cells. Among the tested chemometric algorithms, the OSC-PLS showed the best performance for simultaneous monitoring of Pb, Cd and Zn whose performance was comparable with AAS as reference method. The results of this work showed that chemometrics has a great potential for assisting instrumental techniques to develop accurate novel methods which have very better performance than those instrumental alone. As a new research field for our research group, we are going to continue coupling of chemometric method with instrumental techniques for bioanalytical purposes and definitely, this work will be a bridge to connect the world of chemometricians with the world of bioanalysts.

## Statement

The main idea of this project belongs to Dr. Ali R. Jalalvand and the other authors contributed equally in this project.

## Declaration of Competing Interest

The authors declare that they have no known competing financial interests or personal relationships that could have appeared to influence the work reported in this paper.
